# Using reflection to influence practice: student perceptions of daily reflection in clinical education

**DOI:** 10.1007/s40037-016-0293-1

**Published:** 2016-09-15

**Authors:** Douglas P. Larsen, Daniel A. London, Amanda R. Emke

**Affiliations:** 1 Department of Neurology, Washington University in St. Louis School of Medicine, St. Louis, USA; 2 Department of Orthopedic Surgery, Icahn School of Medicine at Mount Sinai, New York City, USA; 3 Department of Pediatrics, Washington University in St. Louis School of Medicine, St. Louis, USA

**Keywords:** Reflection, Reflection-in-action, Reflection-on-action, Practice-based learning

## Abstract

**Purpose:**

Reflection is a key element in learning from experience, but the impact of most programmes of reflection on daily practice remains unclear. We investigated students’ perceptions of adding a daily written reflection assignment to a clinical rotation.

**Methods:**

Third-year medical students on a single two-week rotation completed daily reflections analyzing their performance. Programme evaluation used a 33-question anonymized survey. Quantitative data were summarized and qualitative responses coded for recurring themes.

**Results:**

Twenty-six students completed the survey (90 % response rate). Eighty-five percent of students felt that the daily reflections had a positive impact on their learning from clinical experience. Seventy-seven percent of students reported that the programme changed their awareness of their thoughts and actions, and 80 % felt that it improved their recall of experiences. A greater sense of mindfulness and focus on self-improvement were major themes that emerge from students’ descriptions of the role of daily reflections in their learning.

**Conclusion:**

Overall, daily reflections demonstrated a positive learning influence. This exploratory study suggests students may benefit from more frequent, short reflections as opposed to more typically spaced reflective assignments.

**Electronic Supplementary Material:**

The online version of this article (doi:10.1007/s40037-016-0293-1) contains survey questions that are available to authorized users.

## What this paper adds

- Often written reflections are used as a form of social inquiry rather than a means of monitoring and changing daily practice. Studies have not typically examined the role of frequency in determining how reflection can be used to influence performance. We show that brief daily written reflections influence students’ learning from experience, increase their awareness of their thoughts and actions, and increase their perceived recall of experiences. The frequency of written reflections should be an important consideration in developing reflection programmes to influence daily practice.

## Introduction

Learning from experience is foundational for physician professional identity formation [1]. Reflection is a key part of most experiential learning theories [2, 3]. However, reflection is rarely a formal part of the daily work of physician education. Typically, formal written reflection is done on an intermittent basis, and learners are asked to describe and analyze major events [4]. In these settings, reflection is used more as a form of social inquiry rather than as an analysis of practice [5]. While these activities are powerful in helping learners to create meaning from their experiences, they are external to the workflow of the learner and do not address the potential for reflection to inform an individual’s daily actions. In fact, the self-monitoring that alters practice occurs on a smaller timescale as practitioners gain increased consciousness of and modulate their moment-to-moment activities [6, 7]. These two distinct types of reflection have been referred to as reflection-on-action, in which individuals think back on past events, and reflection-in-action, in which individuals are aware of and process events as they happen [6, 7].

Reflection-in-action has been shown to be an important aspect of clinical expertise [8]. Furthermore, the timely self-monitoring of knowledge and abilities is also more accurate than broad self-assessment, thus making it a key aspect of self-regulated learning [9, 10]. While some investigators have developed microanalytic techniques to evaluate in real-time how students formulate plans and monitor actions, these approaches are not practical in most education settings [11]. However, the question arises whether frequent reflection-on-action can influence reflection-in-action by priming the learner to become more aware of their thoughts and actions in the moment. Also, research has shown that the repeated recall of facts leads to their improved retention in memory [12, 13]. Reflection involves the recall of experience. Therefore, repeated, frequent reflection may improve the memory of experiences as well. The challenging question that educators continue to face is how to encourage and optimize this type of situational awareness and monitoring.

We investigated if and how daily written reflections influenced students’ perceptions of learning from experience in a clinical rotation. While daily reflections have been used in education programmes of some health professions such as nurse-midwifery,[14] such frequent written reflection is not common in medical education. We looked specifically at how daily written reflection influenced medical students’ awareness of their thoughts and actions (reflection-in-action) as well as their memory of their experiences. Because the role of supervisors has been shown to be important in learning from reflection,[4] we also asked about students’ perceptions of the feedback that they received. Finally, we looked at the acceptability of this form of intensive reflection for students.

## Methods

### Clinical setting

Students are required to take the neurology clerkship as part of their third-year medical school experience. Within that clerkship, students could select the two-week paediatric neurology rotation from among various other clinical services. The written daily reflections programme was incorporated into the paediatric neurology rotation as a required element for students on the rotation, although it was not graded. Students’ grades were determined by the evaluations from their clinical team and were not set by the paediatric neurology rotation director. Twenty-nine students (approximately 24 % of our total annual medical school class) participated in the daily reflections programme from October 2010 to June 2011.

### Procedures

Students were asked at the beginning of the rotation to set learning goals. On a daily basis, they were then required to write a paragraph in which they analyzed their performance and experiences and established plans for how they would act on what they had learned. Students reflected on a variety of topics including things such as challenging cases, their ability or inability to use new physical exam techniques, their reactions to clinical situations, and how decisions were made for their patients. At the end of each week, students submitted their reflections for written feedback from the rotation director (DPL). Feedback focused on helping students to recognize opportunities that may have been missed or encouraging students to develop a more specific, actionable plan based on their reflection.

### Survey

In September 2011, students who had participated in the daily reflections programme during the study period were invited to take an anonymous 33-question electronic survey. Questions were designed to explore the various aspects of students’ experiences with the daily reflections and their opinions on how the programme was implemented. A survey was used so that information could be gathered from all participants in the programme in order to characterize the entire cohort. The survey was developed by the rotation director (DPL) and reviewed by other faculty members for clarity. Those who participated in the survey were compensated with a $ 25 Amazon.com gift card. The study was reviewed and granted exempt status by the Washington University in St. Louis Institutional Review Board.

The survey consisted of both quantitative and qualitative questions (see Appendix, available at: doi:10.1007/s40037-016-0293-1). For quantitative questions answered with Likert-type responses, answers were collapsed into positive and negative categories and percentages were calculated to summarize the results. For all other questions, both quantitative and qualitative content analysis were used to summarize the results [15]. With questions in which written answers could be categorized dichotomously (e. g. daily reflections helped with recall of experiences or did not help with recall of experiences), the results were assigned a category, tallied, and then percentages were calculated. For open-ended questions and students’ comments, responses were coded using a constant comparative methodology by three coders (all authors). The first seven surveys were coded to identify emerging concepts. After these codes were reconciled by discussion among the coders, the following six surveys were coded. After another round of reconciliation, the remaining 13 surveys were coded. These codes were then reconciled as well. All previously coded surveys were reviewed and re-coded as the codes evolved. One final round of reconciliation was used to review all surveys together and to group codes into major themes. Due to space limitations, only the questions that yielded the most broadly applicable results are reported below. A code for each student is included in parentheses after their quote.

## Results

### Response rates

Twenty-six students responded to the survey (90 % response rate). Fifty-four percent of respondents were female. For the data reported below, all students answered the question and provided comments unless otherwise indicated.

### Learning from experience through daily reflection

Students felt that the daily reflections influenced how they learned on the paediatric neurology rotation (see Table 1 for summary of quantitative results). Eighty-five percent of students felt that daily reflections improved their ability to learn from experience to some degree. Daily reflections captured key learning experiences during the rotation for 79 % of students who answered the question (two did not respond to this question).

**Table 1:** Summary of quantitative survey results

Survey item	Percentage of students who gave these responses (%)	Total number of students who provided an answer to the question (*n* = 26)
*Daily written reflections ...*		
Helped with learning from experience	85	26
Captured key learning experiences	79	24
Improved clinical performance	54	26
Differed from spontaneous reflections on other rotations	85	26
Increased awareness of thoughts and actions	77	26
Increased recall of experiences	80	25
*Students who ...*		
Felt that feedback was helpful	65	26
Preferred less frequent reflections	54	26
Felt that daily reflections should continue to be required	72	25

In answering an open-ended question in which students were to describe the role the daily reflections played in their learning during the paediatric neurology rotation, three themes emerged from their descriptions: 1. increased attention to thoughts and processes, 2. additional focus on self-assessment and improvement, and 3. negative feelings about being required to write reflections. Students’ descriptions often contained elements of both reflection-in-action as well as reflection-on-action. *‘Not every day was ground breaking; however, I appreciated how it made me reflect on each day’s events. I had to be present in the moment, in order to effectively analyze what I experienced’ *(ST5). Students often referred to a focus on their thoughts. *‘When I remembered to actively think about my learning and the processes in front of me the reflections served an important way to consolidate my experiences at the end of the day’ *(ST7). In regards to self-assessment, students often commented on the role that the reflections played in identifying deficiencies and helping to create plans for change. As one student said, *‘The most important thing about the daily reflections is that it forced me [to] critically and objectively evaluate my weaknesses and mistakes, which is often overlooked in education. That way I could make plans for improvement and watch my improvement over time’ *(ST2). Despite these benefits, some students felt that writing the reflections was laborious and took time away from other activities. *‘I felt it was a chore to write down something about every day. I usually did it early morning next day so that I wont [sic] forget the details of the day. I always forced myself to write something about the day even though the previous day might have been boring’ *(ST9). While these three themes emerged in response to a specific question, they also resonate through the comments students provided to elaborate on the quantified questions outlined below.

Students also described the effect of the reflections programme on their clinical abilities. Fifty-four percent of students thought that the daily reflections changed their clinical performance (two students did not provide comments clarifying their response). For those students who did feel that the reflections influenced their clinical performance, the reflections helped them to increase their focus on areas of improvement, to make plans, and to track those plans. *‘It also provided a forum for me to write about challenges I faced. I made sure to have an action plan when a problem was identified in order for me to make improvements’ *(ST5). For those students, the reflections sometimes took on the form of goal setting. *‘I felt that holding myself accountable to my goals made me more motivated to make the most of my days than I otherwise would have. I also did not have to think about what it was I wanted to learn or do for that day; I already had those objectives clear in my mind from my reflections the previous day’ *(ST13).

Written reflections as part of this programme seemed to be distinct from other learning experiences for the students. Eighty-five percent of students felt that the reflections they wrote about as part of the daily assignment were different than reflections that spontaneously arose on other rotations. The major difference identified was that their daily written reflections tended to focus more on analysis and self-assessment. *‘It provided a groundwork and consistency in understanding the WHY, as opposed to WHAT happened. For example, a missed procedure in my ordinary reflections reminds me that it was missed, and to do better next time. With these reflections, it required me to probe why I missed the procedure, and how can I set myself up in order to do better next time’ *(ST18, emphasis in the original). In some cases, though, the act of writing daily reflections felt more strained than natural reflections. *‘My reflections on the peds neuro rotation were more thought out and rational; sometimes even forced. I sometimes felt that I had to derive meaning or ’reflect‘ on experiences that I otherwise would not have’ *(ST15).

### Effects of daily reflection on reflection-in-action

Daily reflections were perceived to influence students’ thoughts and actions during the rotation. Seventy-seven percent of students reported that the daily reflections changed awareness of their thoughts and actions. Three students did not provide comments elaborating on their response. One student stated, *‘Once I understood the purpose behind the exercise I tried to frequently remind myself to think about what I was doing. Realizing taking an active introspective role in my clinical decision making was the next step to becoming a good physician this is something that I’ve tried to continues [sic], though I’m no longer writing the reflections’ *(ST7). Students also commented that the accountability of writing each day caused them to pay more attention to the events of the day. However, there was a tension for some students about whether this constant awareness was also distracting. *‘When you know that you have to have at least one experience each day that’s worth writing about, I think it’s natural to spend the day evaluating each of your experiences to see if they would work for the assignment. So in general, I think that I was more aware of my actions, but I’m not sure if this was a good kind of awareness, or a distraction from learning’ *(ST22).

### Effects of daily reflection on the memory of experiences

The daily reflections seemed to influence students’ perceived retention of what they learned on the rotation. Eighty percent of students who answered the question felt that the daily reflections improved their recall of their experiences (one did not answer). Students commented on how the act of writing seemed to be a key mechanism for achieving this greater recall. *‘I think by writing things down and forcing me to remember/think about things that happened has helped me remember some of the lessons during the rotation’ *(ST21). Students often remarked that clinical skills were what they most remembered from their reflections. *‘I remember a great deal more of the situational context in which clinical decisions were made and how I had the chance to gain experience with several different physical exam skills’* (ST14).

### Role of feedback in learning from reflections

Feedback on their reflections played an important part in how students used their reflections. Sixty-five percent of students felt that feedback on their reflections was useful. Students often commented how the feedback led to changes in how they wrote their reflections and that the feedback often caused them to be more analytical of their performance. As one student commented, *‘It [feedback] helped me to think about the things I was reflecting on and maybe see them in a different way or dig a little deeper so that I can learn more from an experience.’ *(ST16).

### Acceptability of daily reflections

Students invested substantial work into their reflections with 92 % of students reporting that they gave moderate to significant effort in writing their reflections during the rotation. On average, students reported spending 27 min a day (range 7–90 min) writing their reflections. While 46 % of students felt that their perceptions of the daily reflections programme did not change over the course of the rotation, 39 % had a more positive view of the reflections over time, and 16 % had a more negative view over time.

Students also had mixed responses with regards to the perceived influence of the daily reflections on their view of the paediatric neurology rotation. Forty-two percent of students had a more positive view of the rotation because of the daily reflections. Thirty-five percent felt that the reflections either did not change their view of the rotation or they had mixed feelings, and 23 % felt that the daily reflections caused them to have a more negative view of the rotation.

Students were split on their feelings regarding the frequency of the reflections. Fifty-four percent would have preferred less frequent reflections. In fact, many felt that writing reflections less frequently would allow them to focus on more meaningful experiences.* ‘I think less frequent reflections would be more useful as you could reflect on a broader set of experiences within the rotation. The daily humdrum is not always sufficient for a meaningful reflection’ *(ST10). However, other students felt that less frequent reflections would diminish their efficacy. *‘I think it would have been more difficult to nail down details of each experience if they were less frequent’ *(ST21). Another student stated, *‘I don’t think it would be as helpful [if reflections were less frequent], as many of the reflections come best when describing very specific, minute details. Even 48 h later, many of these details became obscured to me, as evidenced by the time or so when I missed a reflection one day and wrote double the next’ *(ST18).

The vast majority of students did not continue to write reflections after they completed the paediatric neurology rotation. The most common reason cited for not continuing written reflections was a lack of time. Seventy-two percent of students who answered the question felt that the daily reflections should continue to be required on the paediatric neurology rotation (one student did not answer).

## Discussion

We found that the majority of students felt daily reflections improved their learning from experience during the rotation. Students described the daily reflections as a means of increasing their attention to their thoughts and processes and focusing their self-assessment. These findings suggest that the daily reflections programme largely accomplished its intended effects of influencing students’ reflection-in-action and self-monitoring in order to enhance learning from experience.

Daily reflection creates a closed-feedback loop in which the learner is able to consider the circumstances of their actions, make plans for how to alter performance, and then follow-up on the outcomes. This approach to closed-loop training in which stimulus, response, and outcomes are tightly associated, monitored, and altered has been investigated in the very basic neural networks of learning [16]. On a larger scale, this same approach is also critical to learning from complex real-life experiences. Reflections broadly spaced over time seem unlikely to provide the same tight feedback loop provided by daily reflections.

Interestingly, the majority of students also felt that daily reflections increased their recall of learning experiences. While objective measures of retention would be ideal, the individualized nature of each student’s experiences and memories makes this almost impossible. In the domain of learning factual knowledge, repeated retrieval of information from memory has been shown to be a powerful tool to generate long-term retention [12, 13]. Since reflection also requires experiences to be retrieved and processed from memory, frequent reflection could increase the retention of experiences and what is learned from those experiences. Indeed, even the mental creation of events that never happened can create false memories of those ‘experiences’ [17]. Our findings with this current study are merely suggestive and require further investigation and confirmation.

Students in our study often expressed that feedback was an important part of their learning from the daily reflections. A growing number of studies demonstrate the importance of supervisor involvement of learner’s self-regulated learning, whether that is through reflection, goal-setting, or engagement in self-directed reading [4, 18, 19]. Feedback on the process of reflection seems to have a greater impact on improving reflection than simply providing feedback on the content of reflection [20]. As educators develop their reflective programmes, careful consideration must be paid to the support that learners receive in writing their reflections.

Our study presents an intensive form of reflection requiring students to write on a daily basis. A slight majority of students reported that they would have preferred less frequent assignments. Many of those students felt that less frequent reflections would have increased their significance. However, other students felt that reducing the frequency of the reflections would prevent them from capturing the nuances of their experiences. Future studies will need to investigate whether other time intervals would provide the optimal balance between affecting daily work and minimizing the load on students. We would note, though, that only 23 % of students clearly had a more negative view of the rotation because of the daily reflections, and approximately 70 % of students felt that the programme should continue to be required.

Our study has important limitations. First, our study consists of a relatively small sample size from a single clinical rotation. Because of this, our findings may not generalize to other populations or other clinical education settings. That said, our survey has a very strong response rate and, therefore, gives a thorough representation of nearly all the students who participated in the programme. Our study is exploratory and serves as a proof of concept. The thorough characterization of a small population in this way provides a foundation for further expansion and additional studies.

Using a survey temporally removed from the actual events of the clinical rotation raises the potential for recall bias. While this is a risk, an important part of our research question was to identify if frequent reflection influences students’ sense of how they retained experience. Therefore, time had to pass for them to make such a judgment. Allowing time to pass also allowed students to compare their experiences using the daily reflections with other rotations’ clinical learning experiences.

Finally, our outcomes are based on student self-report and perception. These outcome measures are intrinsic to the study of individualized learning from idiosyncratic experiences. Unfortunately, other broad, external, and quantifiable outcomes such as grades, test scores, or measures of skills such as physical examination techniques are not necessarily aligned with nor sensitive to the day-to-day learning of students. Importantly, our survey did include extensive opportunities for students to describe their thinking and experiences. This qualitative data provides a much richer source of information about students’ learning experiences with the reflections than if we had only acquired quantitative data. Future studies will need to probe further using other qualitative methods such as semi-structured interviews and focus groups.

## Conclusions

Reflection plays an important role in learning from experience. However, the effect of frequency of reflection has not typically been part of the discourse when considering a programme’s impact on reflection-in-action. We also raise the previously unexplored question of the influence of reflection on the memories of experience. We present this exploratory study to begin that conversation around the role of frequency and memory. While further studies must be done to evaluate the most sustainable and impactful timing of reflection, our results suggest that educators can, and should, consider timescales that are more likely to influence the day-to-day work of learners as they create programmes of reflection and self-regulated learning.

## Caption Electronic Supplementary Material


