# Supplementary material for: Using reflection to influence practice: student perceptions of daily reflection in clinical education
Source: Perspect Med Educ. 2016 Sep 15;5(5):285–91. doi: 10.1007/s40037-016-0293-1 (PMC5035279; doi:10.1007/s40037-016-0293-1)
Supplement: Supplementary file 1 [file 40037_2016_293_MOESM1_ESM.docx]

**Appendix**

**Survey questions**

1. What aspect of the rotation had the greatest impact on your learning? Why?
2. How would you rate the effect of the daily reflections assignment on your learning from your experiences during the paediatric neurology rotation?

☐Majorly detracted from learning from experiences

☐Somewhat detracted from learning from experiences

☐Minimally detracted from learning from experiences

☐No impact on learning from experiences

☐Minimally improved learning from experiences

☐Somewhat improved learning from experiences

☐Majorly improved learning from experiences

1. What types of things did you write about in your daily reflections?
2. Please describe the mixture of description versus analysis in your reflections:

☐Almost all of my reflections consisted of analysis of my performance or ways of personally applying events.

☐Most of my reflections consisted of analysis of my performance or ways of personally applying events with some descriptions of interesting experiences.

☐My reflections were equally distributed between descriptions of interesting experiences and analysis of my performance or ways of personally applying events.

☐Most of my reflections consisted of description of interesting experiences and some analysis of my performance or ways of personally applying events.

☐Almost all of my reflections consisted of descriptions of interesting experiences.

Comments or clarification:

1. On average how much effort did you put into the daily written reflections:

☐Minimal effort

☐Moderate effort

☐Significant effort

1. Describe in detail the role, if any, the daily reflections played in your learning during the paediatric neurology rotation. If the reflections did not play a significant role, then please explain why not. **This is the most important question. Please be frank and thorough.**
2. If other sources of learning had a greater impact on what you learned during the rotation, what were they? Why did they have more impact than the reflections, and how were these sources influenced by the reflections?
3. Did writing the daily reflections lead to any change in how you performed during the rotation? If so, then please explain how. If not, why not?
4. Did writing the daily reflections change your awareness of your thoughts and actions because you knew that you would be writing on them later that day? Why or why not?
5. As you think back on the paediatric neurology rotation, how do the daily reflections affect what you recall that you learned from the rotation?
6. Medical students often have experiences on their rotations that cause them to reflect. How did the content of your written reflections on the paediatric neurology rotation differ from the content of your spontaneous, unwritten reflections on other rotations? How did this affect your learning in each situation?
7. What did you want to accomplish by writing the reflections?
8. The daily reflections were not formally graded or used in grading the clerkship. Were you aware of this? How did that knowledge affect what you wrote? If the reflections were graded or used in grading clerkship performance how would that change your use of the reflections? Grading would be based on the depth and specificity of analysis and evidence that the reflections were being used to improve performance.
9. How well did the reflections capture your learning from the clinical experiences that you encountered during the rotation? Why or why not?
10. How would your experience with the reflections change if they were less frequent, such as weekly or at the end of the two-week rotation?
11. Did the **feedback** you received on your reflections affect your learning? If so how, If not why not?
12. If feedback on your reflections were not provided would that alter the impact of learning from the reflections? Why or why not?
13. Were your reflections directed at yourself, at the faculty audience, or both? How did that influence what you wrote about?
14. Did your perceptions of daily reflections assignment change during the course of the rotation? If so, then how?
15. Did your perceptions of the utility of the reflections change after going through other rotations? If so, then how?
16. Did you continue writing reflections after the rotation? Why or why not?
17. How did the reflections affect your perception of the paediatric neurology rotation overall?
18. On average, how many minutes a day did you spend writing the reflections?
19. Do you consider the reflections busy work? Why or why not?
20. Were the initial directions clear on how to complete the reflections? How could they be changed to be clearer?
21. The current instructions do not direct students to focus on any one activity or skill. If students were instructed to focus on specific competencies (i.e. formation of a differential diagnosis and treatment plan, performing a complete neurological examination, presenting the case, etc.) would that make the reflections more useful and specific or would it limit learning by limiting flexibility?
22. How effective were the example reflections (if you receive example reflections) in helping you to understand the assignment?
23. Would you recommend continuing the reflections as part of the rotation or discontinuing them?
24. How could the reflections be modified to make them a more effective learning tool?

The following questions are important because there is research to support that these features may affect learning from experience and reflection.

1. What was your age at the time of the paediatric neurology rotation?

☐18-25 years old

☐26-30 years old

☐31-35 years old

☐36-40 years old

☐41 years old or older

1. What is your gender?
2. How many months ago did you complete the paediatric neurology rotation?

☐1-3 months ago

☐4-6 months ago

☐7-9 months ago

☐10-12 months ago

1. How many clerkships had you completed prior to doing the paediatric neurology rotation?
